# Undergraduate Engagement in Neutron Scattering as a Pathway to U.S. Competitiveness and a Diverse Scientific Workforce

**DOI:** 10.1021/acsomega.5c09807

**Published:** 2026-01-13

**Authors:** Hillary Smith

**Affiliations:** Department of Physics & Astronomy, 7761 Swarthmore College, 500 College Avenue, Swarthmore, Pennsylvania 19081, United States

## Abstract

Engaging undergraduate students in neutron scattering experiments through beamtime trips, student internships, or class visits is a powerful driver of scientific progress in the United States. These experiences provide hands-on exposure to advanced instrumentation, mentorship from diverse scientists, and insight into large-scale research infrastructure. By contributing meaningfully to proposal development, sample preparation, data collection, and data analysis, students develop confidence, scientific identity, and a sense of belonging, particularly those from underrepresented backgrounds. Early engagement cultivates a skilled and motivated scientific workforce, strengthens the capabilities and mission of national laboratories, and ensures continued U.S. leadership in discovery and innovation.

## Graphical abstract

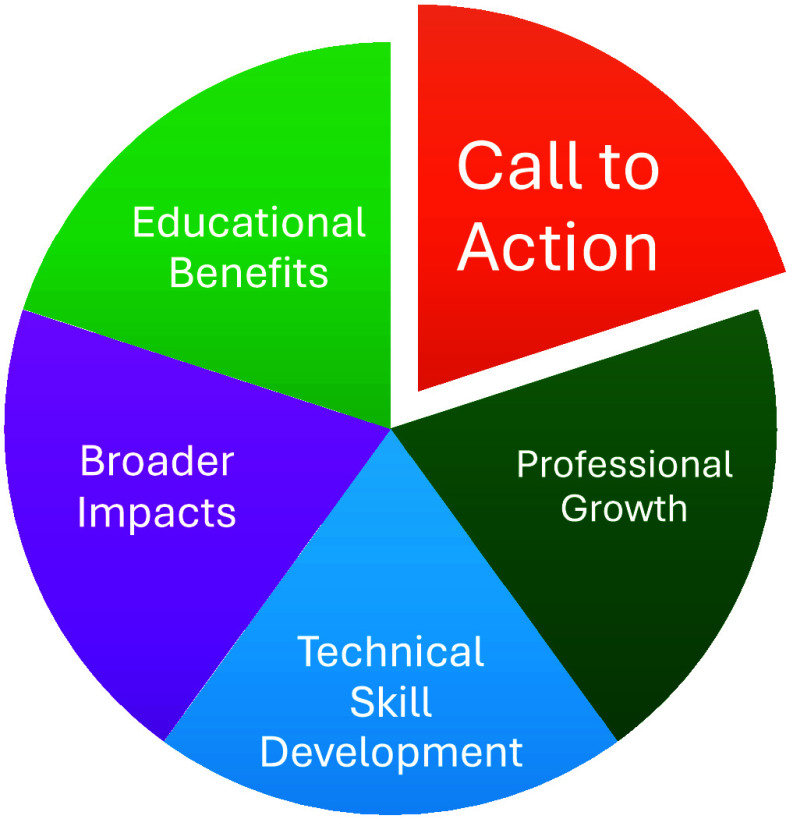

## Introduction

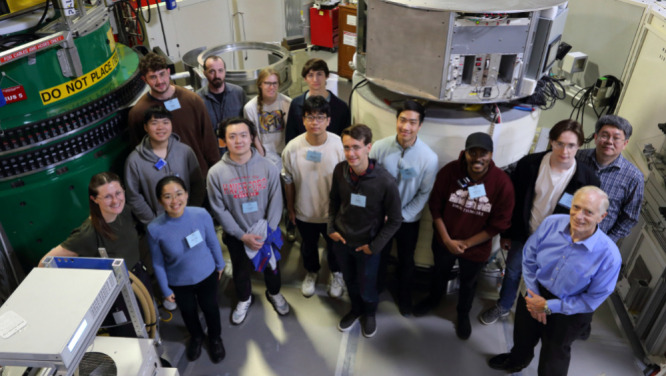



Neutron scattering facilities occupy a unique and vital role within the U.S. scientific ecosystem by providing researchers across the country with access to sophisticated and often one-of-a-kind scientific tools. Researchers access these facilities through user programs that bring faculty, postdocs, and students on site for short “beamtime” trips in which researchers collaborate with facility scientists and staff to perform their experiments. These programs open opportunities not only for established scientists but also for students at multiple stages of their academic journey. Undergraduate students, for example, can join faculty-led research teams for beamtime experiments, participate through national fellowships that place them across laboratory divisions, or engage in one-day educational tours when located near a facility. Unlike many conventional university laboratories, neutron facilities operate on an awe-inspiring scale—housing nuclear reactors or accelerators capable of producing neutrons for dozens of simultaneous experiments. The scope and complexity of these operations and the teamwork of technicians, staff scientists, and PhD researchers who sustain them provide students with a transformative perspective on what scientific research entails. Early exposure to this environment helps undergraduates see themselves as contributors to the larger scientific enterprise and sparks curiosity that drives future discovery.

Despite their central role in advancing U.S. science, national laboratories often remain invisible to undergraduates until graduate school or later.^1^ Opportunities for internships are limited and highly competitive, with especially few positions available in neutron scattering. Broadening undergraduate access through participation in beamtime, faculty-led visits as part of coursework and expansion of internship programs will play critical roles in sustaining U.S. leadership in science. Such engagement not only helps students recognize alternative career pathways beyond academia but also strengthens the pipeline of skilled researchers needed to tackle national challenges.^1–3^

Neutron scattering is unique in that most experiments are performed at large-scale facilities and accessed by a competitive proposal process that selects the most promising science and awards beamtime free of charge (although some university-based reactors offer small neutron sources, the vast majority of experiments can only be performed at large-scale facilities to gain adequate flux and access to specialized instrumentation and sample environments). The three U.S. neutron user facilities that routinely offer open proposal calls for science and technology research are the NIST Center for Neutron Research (NCNR) at the National Institute of Standards and Technology (NIST) and two facilities at Oak Ridge National Laboratory (ORNL), the Spallation Neutron Source (SNS) and the High Flux Isotope Reactor (HFIR). A guide on ‘How to Become a Neutron User’ is provided in Table 1 to suggest a process for PIs new to neutron scattering to become involved. Because neutron resources are extremely limited, the future growth of this field and U.S. competitiveness in neutron scattering depends on cultivating student interest and expertise early. In this way, undergraduate engagement is not just educational enrichment but a strategic investment—fueling scientific progress, diversifying the workforce, and ensuring the vitality of the nation’s research enterprise.

**Table 1:** How to Become a Neutron Facility User^a^

1.	**Sign up to receive facility news.** Facilities regularly distribute information about their proposal calls, workshops, and other information targeted to new users via email. These emails often contain science highlights that may spark ideas for new projects.
2.	**Investigate the facility Web sites**. The facility Web site outlines its science initiatives and current research, offering a helpful resource for generating ideas and identifying questions wellsuited to neutron techniques. Reviewing recent publications and exploring the descriptions of instrument capabilities can also help narrow broad research possibilities to the specific instrument that best matches a new science question.
3.	**Reach out to a beamline scientist.** Send a short description of the scientific problem and how their technique can address it to a beamline scientist. They can arrange a meeting with you to discuss the possibilities for a proposal or forward your request to a colleague. This step is critical in determining the suitability of the project for a neutron experiment and what type of proposal is most appropriate. For example, a brief mail-in experiment may be helpful to obtain preliminary data and provide justification for a multiday general user proposal.
4.	**Prepare a beamtime proposal.** For the best chance of success, access proposal writing tips and watch for proposal writing workshops that are offered by the facility. Share the proposal draft with the beamline scientist to ensure that the experiment plan is feasible and the choice of instrument is appropriate. After the proposal is reviewed, meet again with the beamline scientist to discuss preparing for the experiment or to discuss prospects for revision and resubmission if beamtime was not awarded.

a **Another helpful tip:** Being a first-time facility user can be challenging because it requires extensive collaboration outside our own laboratories. Reaching out to a colleague who is already a facility user and sending a student to “ride along” as a participant in their experiment can be enormously helpful to understand the process from proposal submission, sample preparation, data acquisition, through to data analysis.

## Educational Benefits

Participation in neutron scattering experiments offers significant educational benefits to students and serves as a powerful complement to university-based training. Traditional undergraduate laboratory teaching programs introduce laboratory methods, often by having students replicate established results by using experiments designed decades ago. Some students will also have the opportunity to take their foundational knowledge into research experiences with faculty research groups at their home institutions or through undergraduate research programs at other institutions. The goal of undergraduate research experiences is to transform students’ understanding of science from a set of facts that they learn into a dynamic process of discovery.^4^ This transformation may not always be accomplished if students are working in a single academic group or department and are not exposed to the scale or diversity of the research conducted outside the department.

Participation in neutron scattering experiments exposes students to interdisciplinary teamwork that is the hallmark of scientific advancement at national facilities. Neutron scattering facilities bring together physicists, chemists, biologists, engineers, and computer scientists who contribute different expertise to a shared research goal. For undergraduates who have spent their early science training in disciplinary silos, this exposure can help shape their view of science as an endeavor that requires collaboration across boundaries, with no single discipline holding all the answers. Neutron science thrives on collaboration: an experiment on protein interactions investigates a biological phenomenon, but the instrument is engineered using physics, and the data are analyzed with software developed by computer scientists. Introducing students to collaborative science prepares them for the modern STEM workforce, where the ability to work effectively across disciplines is as essential as technical expertise. Furthermore, students participating in neutron scattering experiments become immersed in the uncertainty and discovery of real science. Unlike classroom laboratories, where experiments are designed to “work”, or undergraduate research projects that are closely managed by graduate students and PIs, neutron scattering experiments often face unexpected challenges.^4^ The research team must navigate these setbacks, instrument calibration issues, sample preparation complications, and inconclusive data, in real time and with a limited amount of beamtime. Observing how scientists adapt, troubleshoot, and maintain focus when facing challenges shows that ambiguity and failure are part of the path to discovery.^5^ Resilience in the face of setbacks is an important skill that students need to thrive in graduate school or future careers in science, and navigating this as an undergraduate student is a valuable experience.^4^

In some cases, participation in experiments on-site is not possible because of travel costs or the timing of experiments that are scheduled during the heart of the academic semester. Mail-in experiments performed remotely by the PI and their research team can also be hugely beneficial in supporting student development. Many of the benefits described here also apply during active engagement in remote experiments, and this participation mode may allow PIs to provide access to a larger team.

## Technical Skill Development

Neutron scattering facilities immerse students in cutting-edge, large-scale experimental science.^1^ Through participation in a beamtime experiment, students gain hands-on experience with sample preparation, instrument calibration, troubleshooting, real-time data analysis, and critical decision making. These activities teach technical competence and introduce students to neutron scattering methods, which connect directly to the foundational curriculum taught in undergraduate chemistry and physics courses.^1,4^ Students have the opportunity to develop advanced computational and data analysis skills while engaging in neutron experiments. Neutron scattering facilities provide access to open data platforms, visualization tools, and modeling software that exceed the scope of undergraduate coursework. While many students will encounter computation in their undergraduate curriculum, the context of the neutron facility helps them see how computing is not just a narrow problem-solving tool but can drive discoveries across scales and disciplines.

It is true that undergraduates cannot take full responsibility for the direction of neutron experiments, leading to critique that the effort and expense to involve undergraduates are better spent on graduate students or postdocs. However, engaging undergraduates in neutron scattering is strategic, both in utilizing the skills of the individual student and in contributing to the preparation of capable researchers for graduate school or the workforce. Undergrads can contribute to experiments by performing routine but essential tasks, such as diluting samples, sealing sample containers, recording metadata, or executing data analysis routines, thereby developing new technical and research skills. Undergrads can also use examples and guidance from their PI to assist in proposal preparation, which is a key aspect of the scientific process and highlights the importance of the careful communication of ideas. To strengthen students’ preparedness, facilities should expand online training modules that faculty can integrate into their coursework or students can complete independently. In doing so, undergraduates can be bolstered from passive observers into active contributors and gain personal satisfaction from their contributions to high-profile research.^6^

## Professional Growth

Undergraduate participation in neutron scattering facilities offers profound opportunities for professional growth that extend far beyond the traditional classroom. By interacting with a wide range of scientists, technical staff, and facility users, students gain first-hand exposure to the diversity of careers available to those with STEM degrees—careers that they may have never encountered at their home institution.^5^ Internships at neutron facilities further enhance this experience by providing direct mentorship from facility scientists and postdocs who can help students envision career paths across academia, government, and industry.^1,4,5^ Table 2 summarizes opportunities available at the NIST Center for Neutron Research and Oak Ridge National Lab.^8–10^ At the NCNR, staff have hosted more than 230 Summer Undergraduate Research Fellowship (SURF) students since 2001, and more than 69 publications from NCNR staff include SURF undergraduate coauthors.^7^

**Table 2:** Internship Opportunities for Undergraduate Students in Neutron Scattering

Internship Program	Eligibility^a^	Summer Opportunities	Academic Year Opportunities	Additional Requirements^a^	Internship Duration	Ref.
DOE Science Undergraduate Laboratory Internships (SULI)	Undergraduate students or recent Associate and Bachelor graduates (within 2 years of graduation)	√	√		Full-time 10 week (summer) and 16 week (AY) program	^10^
DOE Community College Internships (CCI)	Community college students	√	√		Full-time 10 week (summer) and part-time 16 week (AY) program	^10^
ORNL Research Student Internships (RSI)	Undergraduate students or recent Associate and Bachelor graduates (within 2 years of graduation)	√	√		Full-time 10–12 week (summer) or 16–20 week (AY) and part-time 16–20 week (AY) program	^10^
ORNL Technical and Professional Internships (TPI)	Undergraduate students or recent Associate and Bachelor graduates (within 2 years of graduation)	√	√		Full-time 10-12 week (summer) or 16–20 week (AY) and part-time 16–20 week (AY) program	^10^
NIST Summer Undergraduate Research Fellowship (SURF)	Undergraduate students	√			Full-time 9 or 11 week (summer) program	^9^
NIST CHRNS Outreach and Research Experience (CORE) Program	Undergraduate Students or recent Associate and Bachelor graduates (within 1 year of graduation)	√	√		Full-time 9–11 week (summer) and 2–12 month (AY) program	^8^

a All internships listed in the table are only open to U.S. citizens and U.S. permanent residents.

Students engaged in neutron scattering experiments or internships at these facilities come to appreciate the deeply interdisciplinary nature of science. They are often introduced to fields such as biophysics or materials science that are usually not available at undergraduate institutions, with smaller, more traditional departments. This exposure not only broadens their intellectual horizons but also equips them with valuable skills that cross disciplinary boundaries. Ultimately, students leave their experience at neutron facilities better prepared for graduate study, having gained hands-on experience with advanced experiments, a deeper understanding of collaborative science, and the ability to apply principles learned in coursework to new, complex research settings. By supporting the professional growth of undergraduate students, neutron facilities not only strengthen the STEM workforce but also cultivate a pipeline of future researchers, engineers, and technical staff who are already familiar with how neutron facilities operate and have caught the excitement at these facilities.

## Broader Impacts

Including undergraduate students in neutron scattering experiments offers significant broader impacts by shaping the next generation of scientists, supporting workforce development and strengthening the national research infrastructure.^3,11^ Early exposure to neutron scattering encourages long-term engagement with national laboratories and user facilities, helping to cultivate a skilled scientific workforce familiar with the tools, techniques, and collaborative culture of large-scale research environments. Even if students do not utilize these facilities as graduate students, this familiarity may inspire future postgraduate employment opportunities, including technical roles within national laboratories that do not involve traditional research. The NCNR has tracked numerous examples of students who participated in facility internships as undergraduates and have returned to the NCNR, NIST, and other national laboratories.^7^ Thus, facilities benefit from a pipeline of trained, enthusiastic users who can advance both ongoing research and spread the word about these user facilities, which is part of their mission to educate new generations of scientists and engineers.^11^

Participation in neutron scattering experiments also fosters an appreciation for large-scale, government-supported research infrastructure in students and the vital role it plays in advancing the national scientific mission.^3^ For students from smaller or primarily undergraduate institutions (PUIs) who may have limited access to cutting-edge equipment, even touring a neutron scattering facility can be transformative. The National Science Foundation (NSF) Research Experiences for Undergraduates (REUs) are explicitly intended to reach students with limited research opportunities, such as those from small institutions that may not have graduate programs or extensive research laboratories.^12^ Such experiences broaden their understanding of science beyond benchtop experiments, highlighting the scale and impact of modern scientific research.^4^ Moreover, students benefit from exposure to a diverse community of scientists. Through conversations with researchers during a visit to the facility, they can learn about the varied paths, challenges, and personal and professional strategies that lead to success in science. These interactions provide powerful role models and demonstrate that scientific careers are accessible to individuals from all backgrounds.^1,13^ Finally, participation in meaningful experimental work helps students build confidence and develop a sense of belonging in science.^1,4–6^ For undergraduates, particularly those from underrepresented groups, contributing directly to cutting-edge experiments—whether through sample preparation, data collection, or analysis—offers real-time validation of their work.^1,2,13^ This ownership nurtures a scientific identity, reinforcing the likelihood that they will continue to participate in scientific pathways and contribute actively to the research community.

## Call to Action

To fully realize the potential of undergraduate engagement in neutron scattering and its impact on scientific progress, coordinated action is needed from faculty, facilities, and funding agencies. Faculty play a critical role by incorporating neutron scattering modules into their coursework, organizing site visits to national facilities, applying for beamtime, and supporting undergraduates in their participation in remote and on-site experiments with their research groups. Faculty can further enhance opportunities by leveraging their collaborations to secure summer research placements for their students in national laboratories. Neutron scattering facilities can amplify this impact by expanding undergraduate internship programs and developing educational tools tailored to undergraduates for both classroom use and independent exploration. Efforts at facilities to support new users have helped to expand the number of institutions participating in experiments. Targeted outreach to PUIs to support faculty in developing their science case and applying for beamtime is critical to providing opportunities for undergraduate students. Facilities should also engage with institutions to encourage students to apply for internships while also incentivizing staff and postdocs to supervise undergraduates and provide high-quality mentorship. Funding agencies can strengthen these initiatives by prioritizing undergraduate research experiences in grant mechanisms, providing funds for student travel in addition to stipends, and establishing facility-based programs that directly support undergraduates to attend beamtime with their principal investigators.

## Conclusion

The U.S. neutron scattering community urgently needs to sustain a strong pipeline of new researchers because, unlike synchrotron science which benefits from broader facility access and more opportunities for lab-based training, there are only three neutron scattering facilities in the United States with open access to researchers. Thus, engaging undergraduate students in neutron scattering experiments provides profound benefits for both students and the broader scientific community. These experiences offer hands-on opportunities, mentorship, and exposure to advanced facilities that help students—particularly those from underrepresented backgrounds or smaller institutions—build confidence, scientific identity, and a strong sense of belonging in research. Participation fosters long-term engagement with national laboratories and cultivates a diverse scientific workforce capable of advancing discovery and innovation. By appreciating the scale and impact of the government-supported research infrastructure, students gain perspective on the broader scientific mission and the collaborative nature of large-scale experiments. Coordinated support from faculty, facilities, and funding agencies through expanding outreach, developing educational tools, increasing internships, and funding beamtime participation is critical. By acting together, faculty, facilities, and funders can ensure that undergraduate engagement in neutron scattering drives scientific innovation, strengthens the research workforce, and sustains U.S. leadership in the field.

## ABBREVIATIONS

PUI: primarily undergraduate institution
REU: research experience for undergraduates
ORNL: Oak Ridge National Lab
NIST: National Institute of Standards and Technology
NCNR: NIST Center for Neutron Research
